# Systematic Review of the Efficacy and Safety of Shuxuening Injection in the Treatment of Unstable Angina

**DOI:** 10.1155/2022/6650763

**Published:** 2022-02-15

**Authors:** Genhao Fan, Menglin Liu, Jia Liu, Xixi Chen, Yuhong Huang

**Affiliations:** ^1^Tianjin University of Chinese Medicine, Tianjin 301617, China; ^2^Second Affiliated Hospital of Tianjin University of Traditional Chinese Medicine, Tianjin 301617, China

## Abstract

The aim of this study was to systematically review the efficacy and safety of Shuxuening injection combined with conventional Western medicine in the treatment of unstable angina. Randomized controlled trials of Shuxuening injection combined with conventional Western medicine in the treatment of unstable angina were searched by the computer system from PubMed, EMBASE, Cochrane Library, VIP, CNKI, Wanfang Database, and Chinese Biomedical Database since the establishment of the database until June 2020, according to the inclusion and exclusion criteria for the selection of literature, using Rev Man5.3 Meta-analysis Software. The 28 randomized controlled trials were included, with a total of 3,127 patients. Meta-analysis results showed that Shuxuening injection combined with conventional western medicine was effective in improving the clinical efficacy of angina pectoris (RR = 1.23, 95% CI [1.19, 1.27], *P*<0.00001), improvement of ECG (RR = 1.31, 95% CI [1.23, 1.40], *P* < 0.00001), reduction of angina pectoris attack frequency (MD = −1.28, 95% CI [−1.88, -0.67], *P* < 0.0001), duration of angina (MD = −3.36, 95% CI [−3.69, −3.03], *P* < 0.00001), nitroglycerin dosage (MD = -0.39, 95% CI [−0.65, −0.13], *P* = 0.003), C-reactive protein (MD = −2.72, 95% CI [−3.41, −2.03], *P* < 0.00001), BNP (MD = −23.33, 95% CI [−27.87, −18.79], *P* < 0.00001), lower triglycerides (MD = −0.72, 95% CI [−1.05, −0.38], *P* < 0.0001), total cholesterol (MD = −1.39, 95% CI [−1.84, −0.94], *P* < 0.00001), and LDL cholesterol (MD = -1.20, 95% CI [−2.12, −0.29], *P* = 0.01) which is better than that of control group. The effect on raising HDL cholesterol was comparable between the two groups (MD = 0.49, 95% CI [−0.06, 1.04], *P* = 0.08) and the incidence of adverse reactions to differences had no statistical significance (RR = 0.99, 95% CI [0.54, 1.81], *P* = 0.97). The Shuxuening injection combined with conventional Western medicine in the treatment of unstable angina has clear efficacy and a certain degree of safety, so it is recommended for clinical application.

## 1. Introduction

Unstable angina (UA) is an acute coronary syndrome between stable angina and acute myocardial infarction [1], which is mainly manifested as pain in the anterior cardiac region and posterior sternum. Its instability is mainly affected by native coronary artery lesions, and it is characterized by a progressive exacerbation of angina symptoms and prolonged duration of pain. Thrombosis is mainly caused by the rupture of atherosclerotic plaques or the erosion of the mural walls in coronary arteries [2, 3]. The effective treatment of unstable angina can improve the survival rate and quality of life of the patients. Western medicine treatment mainly includes treating stable plaque, using antiplatelet and antithrombotic drugs, reducing myocardial injury brought about by ischemia and reperfusion, and preventing the progression of coronary atherosclerosis. With the popularity of traditional Chinese medicine, it plays a more and more important role in cardiovascular emergencies. The core theory of traditional Chinese medicine is the overall concept and treatment based on syndrome differentiation. It is supposed that it would have an excellent long-term efficacy, complementary with Western medicine treatment, giving full play to each other's advantages and improving the survival rate and quality of life of patients.

The Shuxuening injection is a prescribed herbal medicine of Ginkgo biloba extract; EGb aqueous solution is made by sterilization, with the main effective components such as flavonoid glycosides and terpenoid lactone active substances, including quercetin, galangal, different rat Li Su, Ginkgo biloba lactone, and Ginkgo lactone [4], to relax coronary artery, improve microcirculation, enhance body hypoxia tolerance, and reduce blood viscosity. However, the most clinical studies were clinical experience summary, lacking prospective trial with large sample size, and their pharmacological active components, and pharmacological mechanism still needs more studies to illustrate. In this study, the efficacy and safety of Shuxuening injection combined with conventional Western medicine in the treatment of unstable angina were systematically evaluated to clear the clinical efficacy of Shuxuening injection.

## 2. Methods

The meta-analysis followed the standard set of Preferred Reporting Items for Systematic Reviews and Meta-Analyses (PRISMA). The protocol for this study was registered with CRD42021223531.

### 2.1. Literature Sources

Randomized controlled trials of Shuxuening injection combined with conventional Western medicine in the treatment of unstable angina were searched by the computer system from PubMed, EMBASE, Cochrane Library, VIP, CNKI, Wanfang Database, and Chinese Biomedical Database since the establishment of the database until June 2020. To find out which words are used in China, Shuxuening injection, unstable angina, and any word in English are searched using the search terms “Shuxuening injection” and “Unstable angina.”

### 2.2. Inclusion Criteria

1. Study type: a clinical randomized controlled trial of Shuxuening injection combined with conventional Western medicine in the treatment of unstable angina was conducted.
2. According to “2012 ACCF/AHA Focused Update Incorporated Into the ACCF/AHA 2007 Guidelines for the Management of Patients with Unstable Angina/Non-ST-Elevation Myocardial Infarction,” the subjects met the diagnostic criteria, and there were no restrictions on age, gender, alcohol, and tobacco history.
3. The control group focused on the same conventional Western medicine only. The main drugs were anticoagulation, antiplatelet aggregation, receptor blockers, nitrates, angiotensin-converting enzyme inhibitors (ACEIs), statins, and other drugs, which are used to reduce lipid, while the treatment group was treated with the Shuxuening injection combined with conventional Western medicine.
4. The primary clinical indicators are frequency of angina attacks, duration of angina, nitroglycerin dosage, ECG improvement, and secondary clinical indicators are angina clinical curative effect, blood lipids (triglycerides, total cholesterol, LDL cholesterol, and HDL cholesterol), C-reactive protein, BNP (brain natriuretic peptide), and adverse drug reactions or adverse events.

### 2.3. Exclusion Criteria

The intervention measures did not meet the inclusion criteria, the diagnosis was not clear, and the course of treatment was not clear; the experimental group only used Shuxuening injection or Western medicine compared with the control group, and the control group used other proprietary Chinese medicine; the outcome indicators did not include any other adverse reactions.

### 2.4. Literature Screenings, Data Extraction, and Methodological Quality Evaluation

Two investigators independently read the full text of the literature to extract relevant information. The information extraction included the basic information of the literature, intervention methods, risk of bias assessment (type of study design, randomisation method, allocation concealment, blinding, completeness of data information, and outcome reporting), relevant outcome indicators, and adverse effects. When the two parties disagreed on the inclusion of the literature, the discussion was referred to a third party to judge the final literature for information extraction. The included literature was evaluated against the Cochrane Handbook [5] risk of bias assessment tool for clinical randomised controlled trials with entries on randomisation methods, allocation concealment, blinded implementation, outcome data completeness, selective reporting, and other sources of bias.

### 2.5. Statistical Treatments

RevMan 5.3 Software was used only for the statistical analysis of the included literature review on research data. If significant heterogeneity was found in the experimental results (*I*^2^ ≥ 50%), the random-effects model was used only for the meta-analysis. If the experimental results showed good homogeneity (*I*^2^ < 50%), the fixed-effects model was used for the meta-analysis. The risk ratio (RR) was used as the combined statistic for the meta-analysis, while the mean difference (MD) was used as the statistic for the meta-analysis. If more than 10 references were included in a specified outcome indicator, a funnel plot was used to analyze whether there was publication bias.

## 3. Results

### 3.1. Literature Retrieval

**Figure 1:**
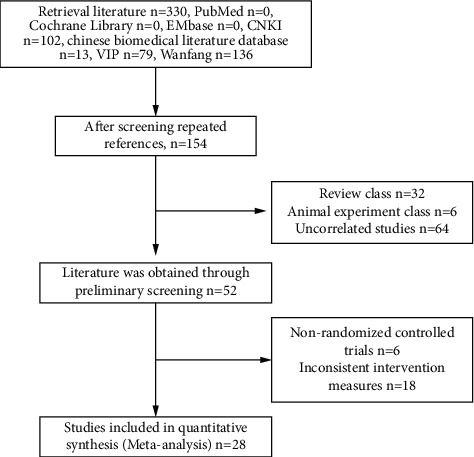
Document selection process and results.

A total of 330 references were retrieved. The titles retrieved from various databases were imported into NoteExpress 3.2.0 Software. A total of 28 titles were finally included after screening [6–33]. The literature screening flow chart and results are shown in Figure 1.

### 3.2. Basic Characteristics of Literature Research

A total of 28 references [6–33] were listed, with a total of 3127 patients. The number of cases in the experimental group was 1584 and that in the control group was 1543. The results are as follows: twenty-five [7, 9–21, 23–33] articles observed the clinical efficacy of angina, five [8, 10, 26, 27] articles observed the frequency of angina, five [6, 8, 10, 13, 27] articles observed the duration of angina, three [17, 22, 27] articles observed the amount of nitroglycerin, 13 [7, 12, 14–16, 20–23, 29, 31–33] articles observed the ECG improvement, and three [11, 16, 29] articles observed the blood lipid. C-reactive protein was observed in 9 [8, 10, 11, 13–16, 18, 26] papers, brain natriuretic peptide was observed in 4 [8, 10, 13, 18] papers, and adverse reactions were observed in 16 [7, 13, 16, 19–25, 27, 29–33] papers, as shown in Table 1.

### 3.3. Quality Evaluation of Included Literature

**Figure 2:**
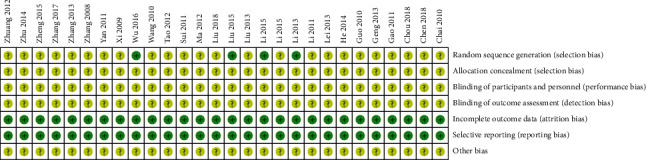
Percentage of projects included in the study that produced a risk of bias.

The works of literature included in this systematic review were evaluated using the Cochrane Handbook. A total of 28 studies were included, 4 studies [10, 12, 13, 18] were grouped using the random number table method, the remaining 24 studies mentioned randomisation but did not specifically describe it, none of the included literature mentioned allocation concealment or blinding, none of the included literature had case loss or dropouts, all studies had complete data, and specific evaluation information on risk of bias is shown in Figure 2.

### 3.4. Meta-Analysis Results

#### 3.4.1. Frequency of Angina Attack

**Figure 3:**
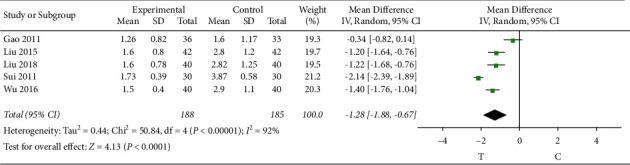
Meta-analysis of angina frequency.

There were 5 articles [8, 10, 13, 26, 27] that observed the frequency of angina, and the heterogeneity test showed that there was significant heterogeneity among the results of studies (*P* < 0.00001, *I*^2^ = 92%); therefore, a random-effects model was used for the meta-analysis, and the results showed a statistically significant difference (MD = −1.28, 95% CI [−1.88, −0.67], *P* < 0.0001), suggesting that the Shuxuening injection combined with conventional Western medicine in patients with lower frequency of angina attack effect is better than that in the control group, as shown in Figure 3.

#### 3.4.2. Duration of Angina Attack

**Figure 4:**
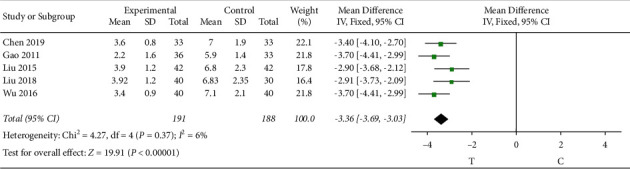
Meta-analysis of duration of angina.

There were 5 studies [6, 8, 10, 13, 27] that observed the duration of angina attack. The heterogeneity test (*P* = 0.37, *I*^2^ = 6%) indicated that there was small heterogeneity between the studies; therefore, a fixed-effects model was used for the meta-analysis, and the results showed a statistically significant difference (MD = −3.36, 95% CI [−3.69, −3.03], *P* < 0.00001), suggesting that the Shuxuening injection combined with conventional Western medicine in patients with reduced duration of angina attack effect is better than that in the control group, as shown in Figure 4. 

#### 3.4.3. Nitroglycerin Dosage

**Figure 5:**
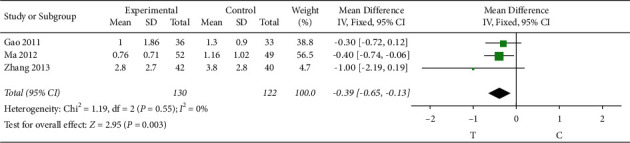
Meta-analysis of nitroglycerin dosage.

3 studies [17, 22, 27] observed the dosage of nitroglycerin. The heterogeneity test (*P* = 0.55, *I*^2^ = 0%) indicated that there was small heterogeneity between the studies; therefore, a fixed-effects model was used for the meta-analysis, and the results showed a statistically significant difference (MD = −0.39, 95% CI [−0.65, −0.13], *P* = 0.003), suggesting that the Shuxuening injection combined with conventional Western medicine in patients with less dosage of nitroglycerin effect is better than that in the control group, as shown in Figure 5.

#### 3.4.4. Improvement of ECG

**Figure 6:**
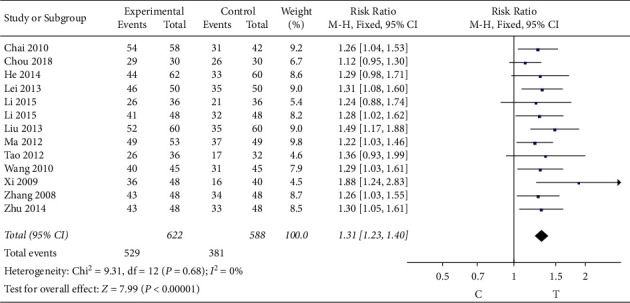
Meta-analysis of the improvement of electrocardiogram.

13 studies [7, 12, 14–16, 20–23, 29, 31–33] observed the electrocardiogram improvement in the study literature. The heterogeneity test (*P* = 0.68, *I*^2^ = 0%) indicated that there was small heterogeneity between the studies; therefore, a fixed-effects model was used for the meta-analysis, and the results show that the difference is statistically significant (RR = 1.31, 95% CI [1.23, 1.40], *P* < 0.00001), suggesting that the Shuxuening injection combined with conventional Western medicine in patients with role of the electrocardiogram improvement is better than that in control group, as shown in Figure 6.

#### 3.4.5. Clinical Efficacy of Angina

**Figure 7:**
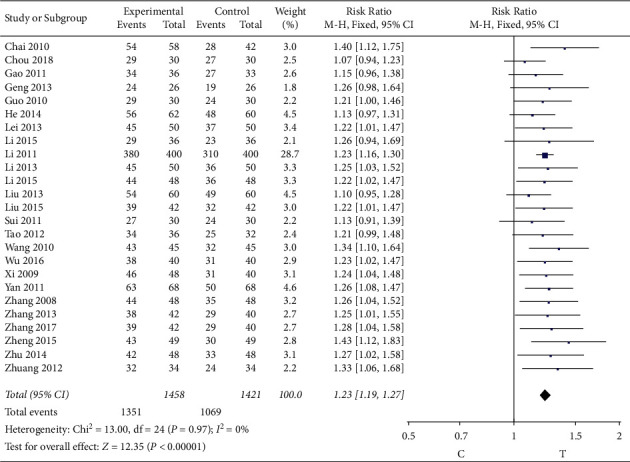
Meta-analysis of clinical efficacy of angina.

25 studies [7, 9–21, 23–33] observed the angina clinical curative effect. The heterogeneity test (*P* = 0.97, *I*^2^ = 0%) indicated that there was small heterogeneity between the studies; therefore, a fixed-effects model was used for the meta-analysis, and the results show that the difference is statistically significant (RR = 1.23, 95% CI [1.19, 1.27], *P* < 0.00001), suggesting that the Shuxuening injection combined with conventional Western medicine in improving patients with angina clinical curative effect is better than that in control group, as shown in Figure 7.

#### 3.4.6. Blood Fat

**Figure 8:**
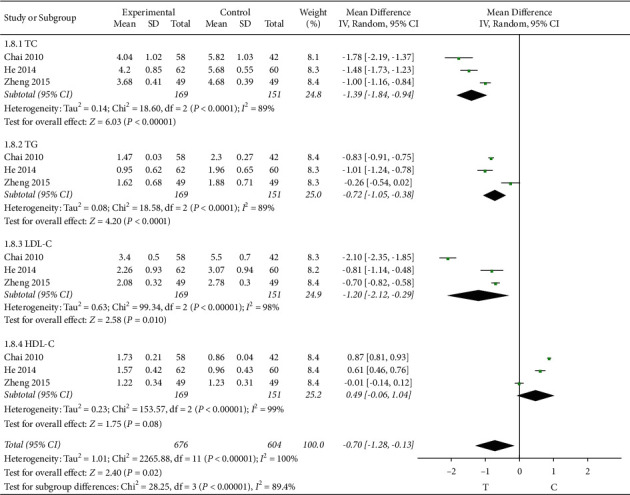
Meta-analysis of blood fat.

3 studies [11, 16, 29] observed the triglyceride (TG), and the heterogeneity test showed that there was significant heterogeneity among the results of studies (*P* < 0.0001, *I*^2^ = 89%); therefore, a random-effects model was used for the meta-analysis, and the results show a statistically significant difference (MD = −0.72, 95% CI [−1.05, −0.38], *P* < 0.0001), suggesting that the Shuxuening injection combined with conventional Western medicine in patients with lower triglycerides effect is better than that in the control group; 3 studies [11, 16, 29] observed the total cholesterol (TC), and the heterogeneity test showed that there was significant heterogeneity among the results of studies (*P* < 0.0001, *I*^2^ = 89%); therefore, a random-effects model was used for the meta-analysis, and the results show a statistically significant difference (MD = −1.39, 95% CI [−1.84, −0.94], *P* < 0.00001), suggesting that the Shuxuening injection combined with conventional Western medicine in patients with lower total cholesterol effect is better than that in the control group; 3 studies [11, 16, 29] observed the low-density lipoprotein cholesterol (LDL-C), and the heterogeneity test showed that there was significant heterogeneity among the results of studies (*P* < 0.00001, *I*^2^ = 98%); therefore, a random-effects model was used for the meta-analysis, and the results showed a statistically significant difference (MD = −1.20, 95% CI [−2.12, −0.29], *P* = 0.01), suggesting that the Shuxuening injection combined with conventional Western medicine in patients with lower LDL cholesterol effect is better than that in the control group; 3 studies [11, 16, 29] observed the high-density lipoprotein cholesterol (HDL-C), and the heterogeneity test showed that there was significant heterogeneity among the results of studies (*P* < 0.00001, *I*^2^ = 99%); therefore, a random-effects model was used for the meta-analysis, and the results showed no significant statistical significance (MD = 0.49, 95% CI [−0.06, 1.04], *P* = 0.08), suggesting that the Shuxuening injection combined with conventional Western medicine had the same effect on raising high-density lipoprotein cholesterol as the control group, as shown in Figure 8.

#### 3.4.7. C-Reactive Protein

**Figure 9:**
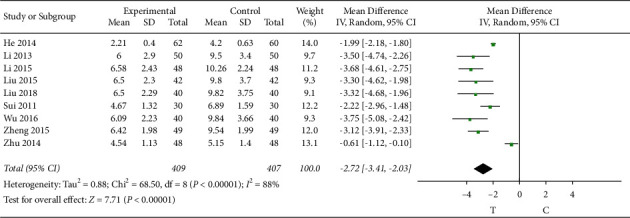
Meta-analysis of CRP.

9 studies [8, 10, 11, 13–16, 18, 26] observed the C-reactive protein, and the heterogeneity test showed that there was significant heterogeneity among the results of studies (*P* < 0.00001, *I*^2^ = 88%); therefore, a random-effects model was used for the meta-analysis, and the results showed a statistically significant difference (MD = −2.72, 95% CI [−3.41, −2.03], *P* < 0.00001), suggesting that the Shuxuening injection combined with conventional Western medicine in improving patients with the role of C-reactive protein is superior to that in the control group, as shown in Figure 9.

#### 3.4.8. Brain Natriuretic Peptide (BNP)

**Figure 10:**
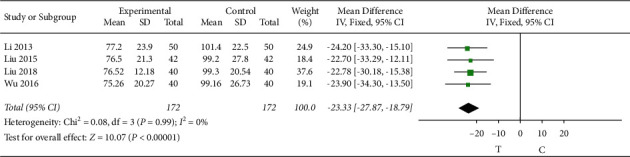
Meta-analysis of BNP.

4 studies [8, 10, 13, 18] observed the brain natriuretic peptide. The heterogeneity test (*P* = 0.99, *I*^2^ = 0%) indicated that there was small heterogeneity between the studies; therefore, a fixed-effects model was used for the meta-analysis, and the results showed a statistically significant difference (MD = −23.33, 95% CI [−27.87, −18.79], *P* < 0.00001), suggesting that the Shuxuening injection combined with conventional Western medicine in patients with reducing effect of brain natriuretic peptide is superior to that in the control group, as shown in Figure 10.

#### 3.4.9. Adverse Reactions

**Figure 11:**
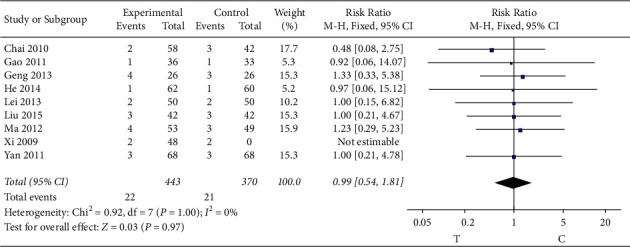
Meta-analysis of adverse reactions.

16 studies [7, 13, 16, 19–25, 27, 29–33] observed the adverse reactions, including 9 documents, as shown in Table 2, and the heterogeneity test (*P* = 1.00, I2 = 0%) indicated that there was small heterogeneity between the studies; therefore, a fixed-effects model was used for the meta-analysis, and the results showed no significant statistical significance (RR = 0.99, 95% CI [0.54, 1.81], *P* = 0.97) and showed quite adverse reactions occurring between the two groups, as shown in Figure 11.

### 3.5. Risk Assessment of Bias

**Figure 12:**
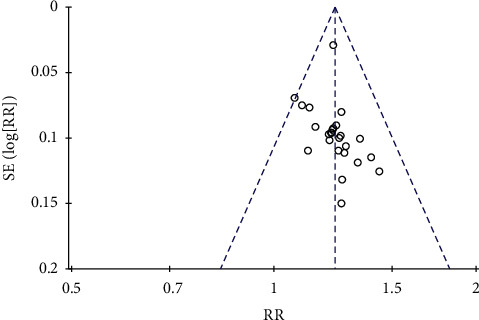
Funnel plot of clinical efficacy of angina.

**Figure 13:**
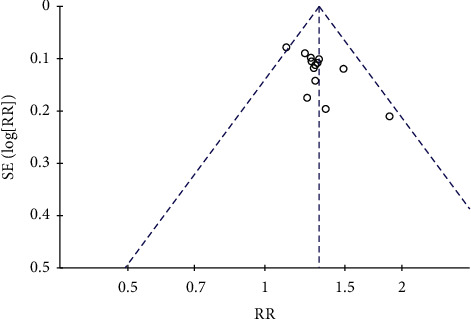
Funnel plot of ECG improvement.

For more than 10 of the included works of literature, the risk of publication bias was assessed. The funnel plot of clinical efficacy and ECG improvement outcome index of angina pectoris was observed. The results showed the incomplete symmetry between left and right, suggesting the risk of publication bias, which may be related to the quality of the included literature and the size of the sample, as shown in Figures 12 and 13.

## 4. Discussions

### 4.1. Research Results

The main purpose of this study is to observe the clinical efficacy of Shuxuening injection in the treatment of unstable angina pectoris. The results showed that the integrative therapy was better than the solo conventional Western medicine in the unstable angina including the improvement of clinical curative effect and the reduction in the frequency of angina, angina duration, dosage of nitroglycerin, CRP, the BNP, lower triglyceride, total cholesterol, and low-density lipoprotein cholesterol. There is no significant difference in the incidence of adverse reactions. The clinical application of the integrative therapy in the treatment of unstable angina has clear efficacy and certain safety, providing more evidence-based evidence for the clinical application of Shuxuening injection in the future.

### 4.2. Research Significance

The Shuxuening injection consists of the active components of Ginkgo leaf extract, which include flavone glycosides and terpenoids, and it can affect oxidation reaction, remove oxide free radical, improve blood flow state, inhibit platelet aggregation, and promote vascular smooth muscle relaxation [34]. The Shuxuening injection with multicomponents and multi-targets is an encouraging complementary to the Western medicine treatment in the unstable angina, especially to improve the survival rate and quality of life of patients. The adverse reactions of the integrative therapy were recorded in detail to help the clinical practice to prescribe.

### 4.3. Limitations of the Study

Our study is to evaluate the efficacy and safety of the integrative therapy in the treatment of unstable angina; meanwhile, it also has the following limitations: firstly, in 28 papers, only four studies were allocated using a random number table, and the rest of the 24 studies just mentioned the randomization without description. There is no mention of allocation concealment and blinding in any of the included literature. All studies have no shedding or withdrawal. Secondly, most of the included studies were single-center randomized controlled studies, and the sample size was small. Only one study had a large sample size of 500 patients, but the calculation method of the sample size was not explained. Thirdly, conventional Western drugs were not described in detail in the included studies, which attributed to the study heterogeneity.

### 4.4. Thinking about the Future

Investigators need to include high-quality clinical randomised controlled trials in future. That is a key factor to improve the level of evidence. For the implementation of specific solutions to clear the random method, estimation of sample size, allocation concealment and blinding, attrition bias, and records of the adverse reactions during the study period should be paid more attention. The clinical study register is important too. TCM treatment should be based on syndrome differentiation, and TCM syndrome types should be specifically stipulated in the inclusion criteria so as to more improve the quality of life and prognosis of patients.

## 5. Conclusion

It is concluded that the Shuxuening injection combined with conventional Western medicine could improve the onset of unstable angina frequency, reduce the dosage of nitroglycerin, and improve ECG, the blood lipid, CRP, and BNP, but the quality of all included studies was low, so the more higher quality of clinical randomized controlled trials will be needed to further demonstrate the validity of Shuxuening injection combined with conventional Western medicine in the unstable angina.

## Figures and Tables

**Table 1:** Literature search.

Study IDs	Sample size		Duration (days)	Intervention		Age		Gender		Outcomes
	T	C		T	C	T	C	M	F	
Chen and Sun 2018 [6]	33	33	14	Basic care + Shuxueninginjection20 ml, ivgtt, qd	Basic care	58.1 ± 2.0	57.8 ± 2.4	—	—	③
Qiu 2018 [7]	30	30	14	Basic care + Shuxueninginjection20 ml, ivgtt, qd	Basic care	72.4 ± 2.3	70.9 ± 2.2	24	36	①⑧⑨
Liu 2018 [8]	40	40	14	Basic care + Shuxueninginjection20 ml, ivgtt, qd	Basic care	58.76 ± 4.50	58.90 ± 4.72	46	34	②③⑤⑥
Zhang 2017 [9]	42	40	30	Basic care + Shuxueninginjection20 ml, ivgtt, qd	Basic care	55 ± 6	53 ± 9	48	34	①
Wu 2016 [10]	40	40	14	Basic care + Shuxueninginjection20 ml, ivgtt, qd	Basic care	61.2 ± 3.1	61.5 ± 3.2	49	31	①②③⑤⑥
Zheng and Li 2015 [11]	49	49	20	Basic care + Shuxueninginjection20 ml, ivgtt, qd	Basic care	—	—	43	55	①⑤⑦
Li et al. 2015 [12]	36	36	28	Basic care + Shuxueninginjection20 ml, ivgtt, qd	Basic care	64.3 ± 1.2	63.1 ± 1.3	39	33	①⑧
Liu 2015 [13]	42	42	14	Basic care + Shuxueninginjection20 ml, ivgtt, qd	Basic care	60.5 ± 3.2	61.3 ± 3.4	49	35	①②③⑤⑥⑨
Liu and Cheng 2015 [14]	48	48	14	Basic care + Shuxueninginjection20 ml, ivgtt, qd	Basic care	59.38 ± 6.22	54.87 ± 4.95	57	39	①⑤⑧
Zhu 2014 [15]	48	48	14	Basic care + Shuxueninginjection20 ml, ivgtt, qd	Basic care	63.8 ± 11.2	64.1 ± 10.8	56	40	①⑤⑧
He et al. 2014 [16]	62	60	28	Basic care + Shuxueninginjection20 ml, ivgtt, qd	Basic care	55.80 ± 3.14	52.80 ± 2.90	63	59	①⑤⑦⑧⑨
Zhang 2013 [17]	42	40	15	Basic care + Shuxueninginjection20 ml, ivgtt, qd	Basic care	54 ± 8	55 ± 8	48	34	①④
Liu and Zhang 2013 [18]	50	50	14	Basic care + Shuxueninginjection25 ml, ivgtt, qd	Basic care	58.7 ± 6.5	57.9 ± 5.3	53	47	①⑤⑥
Geng 2013 [19]	26	26	14	Basic care + Shuxueninginjection20 ml, ivgtt, qd	Basic care	59.5 ± 7.6	58.2 ± 8.3	29	23	①⑨
Lei and Xiao 2013 [20]	50	50	14	Basic care + Shuxueninginjection20 ml, ivgtt, qd	Basic care	56.3 ± 2.6	55.6 ± 2.5	70	30	①⑧⑨
Liu and Wei 2013 [21]	60	60	14	Basic care + Shuxueninginjection10 ml, ivgtt, qd	Basic care	—	—	62	58	①⑧⑨
Ma and Yao 2012 [22]	53	49	10	Basic care + Shuxueninginjection20 ml, ivgtt, qd	Basic care	68.79 ± 10.31	68.12 ± 10.27	67	35	④⑧⑨
Tao and Chen 2012 [23]	36	32	14	Basic care + Shuxueninginjection20 ml, ivgtt, qd	Basic care	—	—	36	32	①⑧⑨
Zhuang 2012 [24]	34	34	14	Basic care + Shuxueninginjection10 ml, ivgtt, qd	Basic care	—	—	41	27	①⑨
Yan 2011 [25]	68	68	14	Basic care + Shuxueninginjection20 ml, ivgtt, qd	Basic care	—	—	76	60	①⑨
Sui 2011 [26]	30	30	14	Basic care + Shuxueninginjection20 ml, ivgtt, qd	Basic care	—	—	36	24	①②⑤
Gao and Feng 2011 [27]	36	33	15	Basic care + Shuxueninginjection20 ml, ivgtt, qd	Basic care	—	—	43	26	①②③④⑨
Li 2011 [28]	400	400	15	Basic care + Shuxueninginjection20 ml, ivgtt, qd	Basic care	58.20 ± 5.90	58.70 ± 6.20	430	370	①
Chai 2010 [29]	58	42	7	Basic care + Shuxueninginjection20 ml, ivgtt, qd	Basic care	64.3 ± 2.3	62.6 ± 1.9	64	36	①⑦⑧⑨
Guo 2010 [30]	30	30	14	Basic care + Shuxueninginjection20 ml, ivgtt, qd	Basic care	—	—	—	—	①⑨
Wang 2010 [31]	45	45	14	Basic care + Shuxueninginjection20 ml, ivgtt, qd	Basic care	—	—	—	—	①⑧⑨
Zhang et al. 2008 [32]	48	48	15	Basic care + Shuxueninginjection20 ml, ivgtt, qd	Basic care	63.8 ± 11.2	64.1 ± 10.8	54	42	①⑧⑨
Xi 2009 [33]	48	40	14	Basic care + Shuxueninginjection20 ml, ivgtt, qd	Basic care	56.3 ± 7.8	55.1 ± 4.5	50	38	①⑧⑨

Note: ①: clinical effect of angina; ②: angina frequency; ③: duration of angina; ④: nitroglycerin dosage; ⑤: C-reactive protein; ⑥: brain natriuretic peptide; ⑦: blood fat; ⑧: improvement of electrocardiogram; ⑨: adverse drug reactions or adverse events.

**Table 2:** Adverse reactions.

Study IDs	Adverse reactions	
	T	C
Liu [13]2015	1 case of nausea, 2 cases of local swelling, and 1 case of mild headache	1 case of nausea, 1 case of local swelling, and 1 case of mild headache
He et al. [16]2014	1 case of mild elevation of alanine aminotransferase (<50 U)	1 case of mild elevation of alanine aminotransferase (<50 U)
Geng [19]2013	Facial flushing occurred in 2 cases and dry mouth in 2 cases	1 case of dry mouth, 1 case of diarrhea, and 1 case of dizziness
Lei and Xiao [20]2013	2 cases of skin rash	2 cases of skin rash
Ma and Yao [22]2012	Three patients presented with nausea and one with abdominal distension	Two patients developed nausea and one had palpitation
Tao and Chen [25]2011	Flush appeared in 3 cases	Flush appeared in 3 cases
Gao and Feng [27]2011	One case had mild headache, head distension, and flushed face	Two patients had mild headache, head distension, and flushed face
Chai [29]2010	Patchy ecchymosis appeared at the injection site in 2 cases	Patchy ecchymosis appeared at the injection site in 3 cases
Xi [33]2009	Flushing and dizziness were observed in 2 patients	Flushing and dizziness were observed in 2 patients

## Data Availability

The data are available from PubMed, EMBASE, Cochrane Library, VIP, CNKI, Wanfang Database, and China Biomedical Database.
